# Exogenous Melatonin Enhances the Yield and Secondary Metabolite Contents of *Prunella vulgaris* by Modulating Antioxidant System, Root Architecture and Photosynthetic Capacity

**DOI:** 10.3390/plants12051129

**Published:** 2023-03-02

**Authors:** Qingshan Chang, Lixia Zhang, Shuangchen Chen, Minggui Gong, Longchang Liu, Xiaogai Hou, Yinfa Mi, Xiaohui Wang, Jianzhang Wang, Yue Zhang, Yiming Sun

**Affiliations:** 1 College of Horticulture and Plant Protection, Henan University of Science and Technology, Luoyang 471000, China; 2 College of Agriculture, Henan University of Science and Technology, Luoyang 471000, China; 3 College of Food and Bioengineering, Henan University of Science and Technology, Luoyang 471000, China; 4 Peony Research Institute, Luoyang Academy of Agriculture and Forestry Sciences, Luoyang 471023, China; 5 Luoyang Greening Management Center, Luoyang 471023, China

**Keywords:** antioxidants, melatonin, photosystem, *Prunella vulgaris* L., secondary metabolites

## Abstract

Melatonin (MT) plays a number of key roles in regulating plant growth and secondary metabolite accumulation. *Prunella vulgaris* is an important traditional Chinese herbal medicinal plant which is used for the treatment of lymph, goiter, and mastitis. However, the effect of MT on the yield and medicinal component content of *P. vulgaris* remains still unclear. In this research, we have examined the influence of different concentrations of MT (0, 50, 100, 200, 400 μM) on the physiological characteristics, secondary metabolite contents, and yield of *P. vulgaris* biomass. The results showed that 50–200 μM MT treatment had a positive effect on *P. vulgaris.* MT treatment at 100 μM greatly increased the activities of superoxide dismutase and peroxidase, the contents of soluble sugar and proline, and obviously decreased the relative electrical conductivity, the contents of malondialdehyde and hydrogen peroxide of leaves. Furthermore, it markedly promoted the growth and development of the root system, increased the content of photosynthetic pigments, improved the performance of photosystems I and II and the coordination of both photosystems, and enhanced the photosynthetic capacity of *P. vulgaris*. In addition, it significantly increased the dry mass of whole plant and spica and promoted the accumulation of total flavonoids, total phenolics, caffeic acid, ferulic acid, rosmarinic acid, and hyperoside in the spica of *P. vulgaris*. These findings demonstrated that the application of MT could effectively activate the antioxidant defense system of *P. vulgaris*, protect the photosynthetic apparatus from photooxidation damage, and improve the photosynthetic capacity and the root absorption capacity, thereby promoting the yield and accumulation of secondary metabolites in *P. vulgaris*.

## 1. Introduction

*Prunella vulgaris* is a perennial herb belonging to the botanical family Labiatae and is widely distributed in temperate and tropical regions such as China, Japan, Korea, Pakistan, Germany, and the United States of America for its diversity of applications [1]. In the south of China, flowers and fresh leaves of this species can be used as ornamental and vegetables, respectively [2]. Importantly, the dried spicas of *P. vulgaris,* Prunellae Spica, are commonly used as standard herbs for mastitis, pulmonary tuberculosis, goiter, and tuberculosis, and an important raw material for herbal tea. Besides, it is considered as an important raw material for herbal tea, as well as the whole plant can be used as medicine in Europe, Taiwan, and China [3]. In the recent years, it has been observed that there is a growing requirement of raw materials for the pharmaceutical and herbal tea industries and for the production of Chinese patent medicines alone, which would need about 5000 tons of Prunellae Spica every year [4]. Therefore, improving the yield and quality of *P. vulgaris* to meet the strong demand for uniform and high-quality raw materials has become an important issue to be solved urgently.

Research has shown that *P*. *vulgaris* contains a variety of secondary metabolites, rich in phenolic acids and flavonoids such as caffeic acid, ferulic acid, rosmarinic acid, hyperoside, etc. [4]. These phenolic acids and flavonoids are products of secondary metabolic processes and are important medicinal components in *P*. *vulgaris*, having antioxidant, anti-inflammatory, antibacterial, antiviral, anti-leukemia, and anticancer activities, as well as neuroprotective and other biological activities; they also play a crucial role in preventing oxidative damage caused by various abiotic stresses [5]. Previous studies have shown that plants could counteract the toxicity of reactive oxygen species (ROS) by activating enzymatic and non-enzymatic systems [6]. ROS can be partially scavenged by antioxidant enzymes such as superoxide dismutase (SOD), peroxidase (POD), catalase (CAT), and ascorbate peroxidase (APX) [7]. Meanwhile, non-enzymatic antioxidants including phenols, flavonoids, polysaccharides, carotenoids, α-tocopherols, and other compounds can work in concert with antioxidant enzymes to remove ROS.

It is well documented that the exogenous application of phytohormones can regulate crop growth and development, increase yield, and improve quality [8,9]. Melatonin (N-acetyl-5-methoxytryptamine) is an indoleamine hormone, a pleiotropic biomolecule, and a broad-spectrum antioxidant which is widely found in animals and plants [10]. Studies have shown that MT acts as the first line of defense against oxidative stress in plants, which in plant cells can efficiently remove reactive oxygen (ROS) or reactive nitrogen species (RNS), which are both signal molecules and toxic substances to plant cells [11]. Furthermore, MT not only directly detoxifies ROS and RNS as antioxidants, but also indirectly promotes the activities of the main antioxidative enzymes and inhibits the activity of pro-oxidant enzymes, as well as the content of some non-enzymatic antioxidants such as phenolic compounds [12,13]. A set of studies showed that MT can increase antioxidant enzyme activities to remove ROS and RNS, and protect plants from oxidative damage caused by various abiotic stresses such as drought [14], salt stress [15], chromium toxicity, and so on [16]. The application of MT increased stress enzyme activities and biomass accumulation during callogenesis in *P. vulgaris* [17]. It also increased the phenolic compounds and antioxidant activities in two *Citrus* cultivars under drought stress [18]. Other studies have shown that MT can regulate root growth by regulating auxin (IAA) synthesis, transport, and signal transduction in an IAA-dependent manner [19,20]. MT can also be involved in the regulation of *Arabidopsis* root growth by sensing N-acylserine lactones secreted by rhizosphere bacteria through melatonin receptors (CAND2/PMTR1) [21,22,23,24]. In cotton (*Gossypium* spp.) and cucumber (*Cucumis sativus*), exogenous MT could activate the functions of root-related hormones and transcription factor pathways, maintain root cell integrity, increase root vigor, and induce lateral root formation [25,26]. The exogenous application of MT increased the chlorophyll content and photosynthetic capacity in plants [17,27], and enhanced the content of non-structural carbohydrates and nitrogen by upregulating sucrose transporters and nitrogen uptake-related genes, promoting its transport to grains, thereby increasing rice yield under low nitrogen conditions [9]. Under adverse weather conditions, MT treatment improved commercial crop yield and quality traits of sweet cherries [28].

Photosynthesis involves two reaction steps, converting light energy into chemical energy (light reaction) and then converting CO_2_ into organic metabolites (dark reaction), which is the basis of crop yield [29]. The photochemical reaction is the engine of photosynthesis, which depends on the coordination of photosynthetic pigments, the photosynthetic electron transport chain, and two photosystems (PSI and PSII) [30]. Recent studies have shown that MT plays a positive role in protecting photosynthetic apparatus from damage under various stress conditions [31]. Yin et al. [32] found that exogenous MT reduced the accumulation of ROS by balancing the distribution of photosynthetic electron flux, facilitated the repair of PSII, and improved the electron transfer ability of PSII donor and acceptor sides in tomato plants under salt stress. Wu et al. [33] found that MT (100 μM) enhanced the electron transport capacity between PSII and PSI, and increased the oxygen-evolving complex activity and PSII activity under low temperature stress. In maize seedlings, foliar application of exogenous melatonin improved the photosynthetic electron transfer efficiency between both photosystems and alleviated the damage of the quantum yield of PSI and PSII under drought stress conditions [14]. However, the role of MT on the PSI and PSII activities of *P. vulgaris* under stress or non-stress conditions remains unclear. Therefore, it is necessary to study the potential role of MT in improving the photosynthetic apparatus.

The identification of MT receptors suggests that MT is a hormone involved in regulating plant hormone crosstalk [11]. MT has been reported to modulate the biosynthesis and signaling of numerous plant hormones, including auxin, gibberellin (GA), salicylic acid (SA), jasmonic acid (JA), ethylene, strigolactones, brassinosteroids, and abscisic acid (ABA) [11,34,35,36]. Especially, MT plays a positive role in the synthesis of secondary metabolites in plants under stress or non-stress conditions [18]. Xu et al. [12] found that MT enhanced the polyphenol content in grape berries, and Coskun et al. [8] found that MT greatly increased the content of secondary metabolites such as rosmarinic acid and caffeic acid in a callus culture of rosemary (*Rosmarinus officinalis*). Bistgani et al. [37] found that exogenous MT promoted the accumulation of total phenolic compounds in the leaves of garden thyme (*Thymus daenensis*) to respond to salinity stress.

Many reports have shown that MT can promote rhizogenesis, seedling growth, antioxidant ability, photosynthesis, secondary metabolite accumulation, yield, and biomass production, etc. in plants [8,37,38]. However, the effects of its exogenous application on the accumulation of dry matter and secondary metabolites in medicinal plants such as *P. vulgaris* have rarely been studied, and little information is available on its effects on photosynthesis by combining PSII and PSI. Therefore, it is necessary to study the effects of MT on antioxidant enzymes, osmotic adjustment substances, root growth, photosynthetic pigments, gas exchange parameters, structure and function of PSII and PSI, yield, and secondary metabolite contents of *P. vulgaris*. This study will provide a reference to unraveling the response of MT on the growth and development in *P. vulgaris*, and offer theoretical evidence for improving the yield and quality in practice.

## 2. Results

### 2.1. Antioxidant Enzyme Activity and Osmoregulation Substance Content

**Figure 1:**
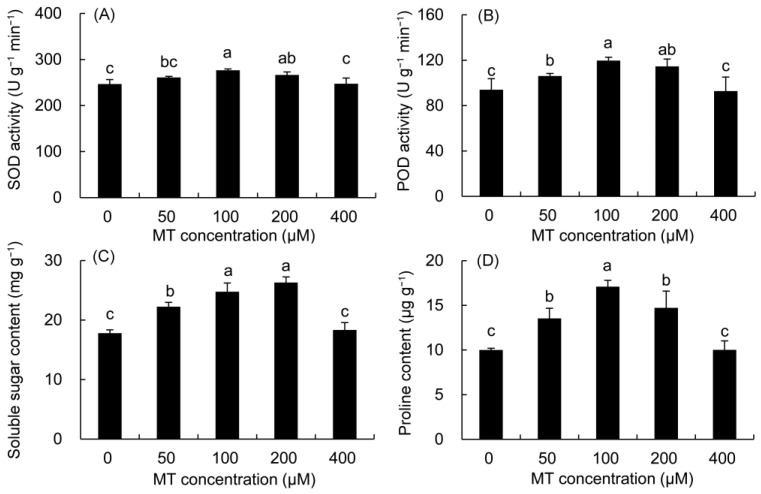
Effects of different concentrations of MT on the activities of SOD (**A**) and POD (**B**) and the contents of soluble sugar (**C**) and proline (**D**) in *P. vulgaris.* MT: melatonin. Data are means ± standard error; different letters indicate significant differences as determined by the Duncan test (*p* ≤ 0.05).

The SOD and POD enzyme activities first increased, but then decreased with the increase in MT concentration (Figure 1). In the 100 μM MT treatment, their activities drastically elevated by 12.08 and 27.35% compared with the control, respectively, reaching the highest value in all MT treatments, followed by the 200 μM treatment.

The contents of soluble sugar and proline were higher in all MT treatments but significantly within the 50–200 μM MT treatments (Figure 1). The soluble sugar content increased by 39.18 and 47.81%, in the 100 and 200 μM MT treatments, respectively compared with the control. The proline contents of the 100 μM and 200 μM MT treatments were 70.88 and 47.16% higher than that in the control, respectively.

### 2.2. REC and the Contents of MDA and H_2_O_2_

**Figure 2:**
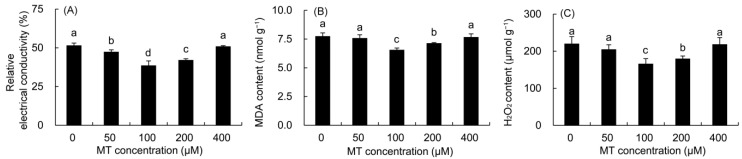
Effects of different concentrations of MT on REC (**A**) and the contents of MDA (**B**) and H_2_O_2_ (**C**) in *P. vulgaris.* MT: melatonin. Data are means ± standard error; different letters indicate significant differences as determined by the Duncan test (*p* ≤ 0.05).

The REC, MDA, and H_2_O_2_ contents were first decreased and then elevated with the increase in MT concentration (Figure 2). The REC was greatly lower in the 50–200 μM MT treatments compared with the control, and the minimum value was recorded in the plants sprayed with 100 μM MT, which was 25.14% lower than that in the control. The MDA and H_2_O_2_ contents decreased markedly in the 100–200 μM MT treatment. Among these, the contents of MDA and H_2_O_2_ decreased the most after the 100 μM MT treatment, which decreased by 15.30 and 24.57% compared to the control, respectively.

### 2.3. The Contents of Chlorophyll and Carotenoid

**Figure 3:**
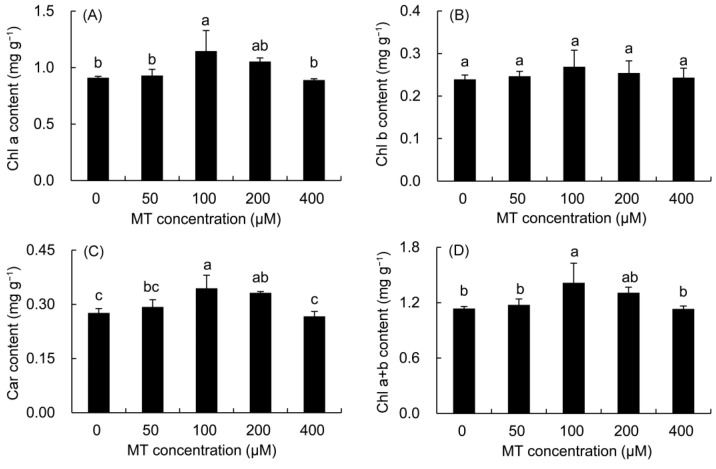
Effects of different concentrations of MT on photosynthetic pigment content of Chl a (**A**) and Chl b (**B**) and Car (**C**) and Chl a + b (**D**) in *P. vulgaris*. MT: melatonin. Data are means ± standard error; different letters indicate significant differences as determined by the Duncan test (*p* ≤ 0.05).

The contents of Chl a, Chl b, Car, and Chl a + b in *P. vulgaris* showed a trend of first increasing and then decreasing with the increase in MT concentration (Figure 3). The increase in Chl a and Chl a + b contents was the highest in the 100 μM treatment, which was elevated by 23.16% and 21.92%, respectively, compared with those in the control treatment. The Chl b content was higher in all MT treatments than in the control treatment, but did not reach a significant level. All MT treatments augmented the Car content of *P. vulgaris* leaves. The 100 and 200 μM MT treatments predominantly enhanced the Car content by 24.74 and 20.18% compared with the control treatment, respectively.

### 2.4. Gas Exchange Parameters

**Figure 4:**
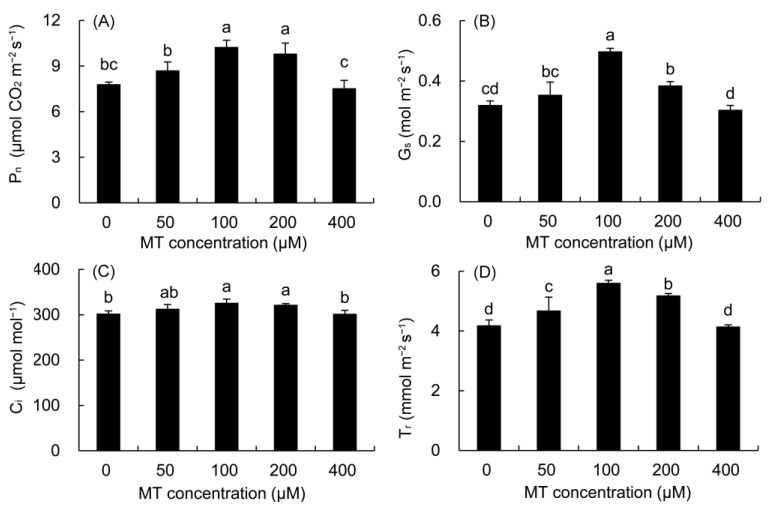
Effects of different concentrations of MT on P_n_ (**A**), G_s_ (**B**), C_i_ (**C**) and T_r_ (**D**) of *P. vulgaris.* MT: melatonin. Data are means ± standard error; different letters indicate significant differences as determined by the Duncan test (*p* ≤ 0.05).

Compared with the control, the P_n_ was higher in all MT treatments except for the 400 μM MT treatment (Figure 4). The maximum was noted in the 100 μM of MT (31.33%), followed by the 200μM MT treatment. Compared with the control, the G_s_, C_i_, and T_r_ of *P. vulgaris* were higher in the 50–200 μM MT treatments and reached significant levels in the 100 and 200 μM MT treatments.

### 2.5. Root Architecture

The root length, total root surface area, and total root volume were higher in the 50–200 μM MT treatments compared with the control (Table 1), and the increase in the 100–200 μM MT treatments all reached a significant level. The root tip number and branch number of *P. vulgaris* was markedly enhanced in the 50–200 μM MT treatments, and the highest value was found in the 100 μM MT treatment. The root tip and branch numbers were considerably lower in the 400 μM MT treatment than in the control treatment.

### 2.6. Chlorophyll Fluorescence OJIP Curve and 820 nm Modulated Reflection

**Figure 5:**
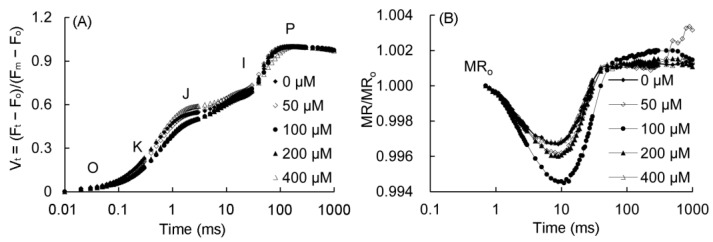
Fast chlorophyll fluorescence (**A**) and 820 nm modulated reflection curve (**B**) of *P. vulgaris* under different MT treatments. V_t_ = (F_t_ − F_0_)/(F_m_ − F_0_): the relative variable fluorescence (V_t_) at any time; O, K, J, I, and P represent different phases in the OKJIP curve. MR/MR_0_: the 820-nm modulated reflection curve; MR is the modulated reflection at different time points; MR_0_ is the MR value of far-red light irradiated at 0.7 ms. MT: melatonin. Data are means ± standard error; different letters indicate significant differences as determined by the Duncan test (*p* ≤ 0.05).

The fast fluorescence OJIP curves and 820 nm modulated reflection curves of *P. vulgaris* are shown in Figure 5. As can be seen from Figure 5A, the K point (*t* = 0.3 ms) and J point (*t* = 2 ms) decreased in the 50–200 μM MT treatment, and the changes were the most obvious in the 100–200 μM treatments. The MR/MR_0_ of the 820 nm reflection curve of *P. vulgaris* began to decrease from 0.7 ms (Figure 5B), and reached to the lowest value after about 10 ms, and increased slowly after 200 ms. The lowest point of the MR/MR_0_ in the 50–200 μM MT treatment decreased, of which the 100 μM group decreased the most, followed by the 200 and 50 μM MT treatments, while the MR/MR_0_ value in the 400 μM treatment had little change.

### 2.7. Fast Chlorophyll Fluorescence Parameters of P. vulgaris

**Figure 6:**
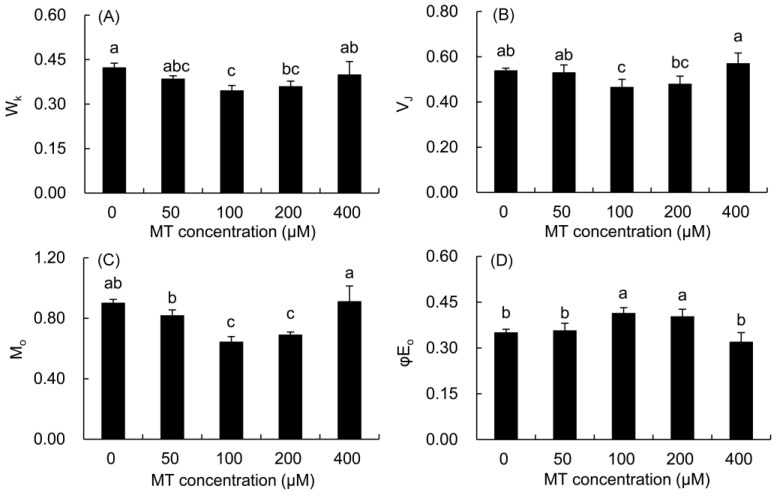
Effects of different concentrations of MT on W_K_ (**A**), V_J_ (**B**), M_o_ (**C**) and φE_0_ (**D**) of *P. vulgaris.* MT: melatonin. Data are means ± standard error; different letters indicate significant differences as determined by the Duncan test (*p* ≤ 0.05).

The normalized fluorescence (W_K_) of the K phase and the relative fluorescence change of the J phase (V_J_) in the MT treatment were calculated to quantify the changes in the K and J phases in the fast chlorophyll fluorescence curve (Figure 6). The values of W_K_, V_J_, and M_0_ first decreased and then elevated with the increase in the MT concentration. The values of the three parameters were significantly lower in the 100 and 200 μM MT groups compared with the control treatment. The value of φE_0_ first increased and then decreased with the increase in the MT concentration. Only the value of φE_0_ was markedly higher in the 100 and 200 μM MT treatments compared with the control treatment.

The ABS/RC, DI_0_/RC, and TR_0_/RC parameters initially decreased and then increased with the increase in the MT concentration (Table 2). Except for the 50 and 400 μM MT treatments, the three parameters were predominantly lower in the 100 and 200 μM MT treatments compared with the control, and decreased the most in the 100 μM MT treatment. Compared with control treatments, the ET_0_/RC of all MT treatments did not reach a significant level. The four parameters RC/CS_m_, ABS/CS_m_, TR_0_/CS_m_, and ET_0_/CS_m_ initially increased and then decreased with the increase in the MT concentration. The above four parameters were all drastically higher in the 100–200 μM MT treatments compared with the control treatment, and the increase was the highest in the 100 μM MT treatment. The DI_0_/CS_m_ in all MT treatments did not reach a significant level compared with the control.

### 2.8. The Function and Coordination of PSII and PSI

**Figure 7:**
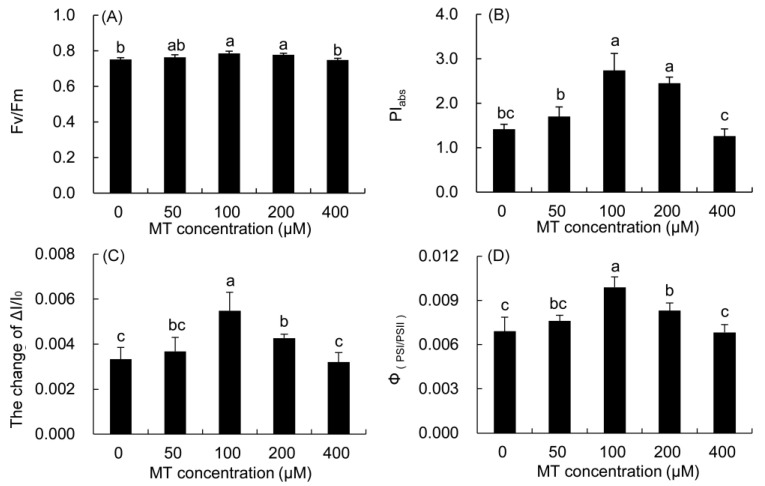
Effects of different concentrations of MT on the function and coordination of PSII and PSI of *P. vulgaris.* Fv/Fm, (**A**); PI_abs_, (**B**); ΔI/I_0_, (**C**) and Φ_PSI/PSII_, (**D**); MT: melatonin. Data are means ± standard error; different letters indicate significant differences as determined by the Duncan test (*p* ≤ 0.05).

The F_v_/F_m_ and PI_abs_ of *P. vulgaris* leaves treated with 100 and 200 μM MT were considerably higher than those in the control (Figure 7), and the increase in the 100 μM treatment was the highest. These two parameters were higher in the 50 μM MT treatment, but lower in the 400 μM MT treatment than the control.

The maximum redox activity (ΔI/I_0_) of PSI and the coordination of PSII and PSI (Φ_PSI/PSII_) in the 50–200 μM MT treatments increased, but decreased in the 400 μM MT treatment compared with the control. The two parameters were markedly higher in the 100 μM MT treatment than those in the control, and the increase was the largest.

### 2.9. Dry Mass of Whole Plant and Spica

**Figure 8:**
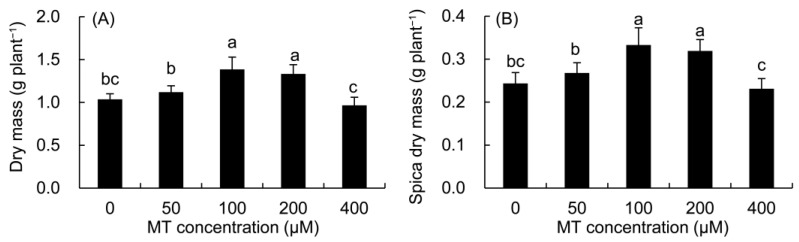
Effects of different concentrations of MT on the dry mass of whole plant (**A**) and spica (**B**) of *P. vulgaris.* MT: melatonin. Data are means ± standard error; different letters indicate significant differences as determined by the Duncan test (*p* ≤ 0.05).

Compared with the control (Figure 8), the dry mass of the whole plant and spica of *P. vulgaris* increased in the 50–200 μM MT treatments, but decreased in the 400 μM MT treatment, and the maximum value was found in the 100 μM MT treatment.

### 2.10. Secondary Metabolite Contents

The contents of total flavonoids and phenolics were drastically higher in all MT treatments compared with the control treatment (Table 3). The contents of total flavonoids and phenolics increased the most in the 100 μM MT treatment. The contents of caffeic acid, ferulic acid, rosmarinic acid, and hyperoside were all higher after the 50–200 μM MT treatments, with the largest increase in the 100 μM MT treatment.

## 3. Discussion

ROS are considered toxic by-products in aerobic metabolism, often produced during chlorophyll biosynthesis and the electron transfer reaction of photosynthesis [39,40]. The SOD enzyme can convert O_2_·^−^ into H_2_O_2_ and O_2_, and the POD enzyme catalyzes H_2_O_2_ to H_2_O and O_2_ [41]. In the present study, pretreatment with MT (100 μM) markedly reduced ROS levels in *P. vulgaris* leaves, which may be due to the fact that MT itself is an antioxidant compound stronger than ascorbic acid and hence might directly react with ROS to stop lipid oxidation. On the other hand, MT treatments improved the activities of SOD and POD, which contributed to the further scavenging of ROS [42,43]; this was also confirmed by the significant decrease in the REC, MDA, and H_2_O_2_ content in *P. vulgaris*. These results were in agreement with previous studies on *Brassica juncea* [44], *Limonium bicolor* [45], and *Momordica charantia* [46] treated with MT. Studies have shown that plants can co-accumulate biochemical solutes such as soluble sugars and proline to regulate the osmotic potential of cells and stabilize the structure of biomolecules to adapt to oxidative stress [4,31]. In particular, soluble sugars and prolines are also involved in the detoxification of ROS in different organelles against abiotic stress [16,47]. In this study, MT treatments increased the content of soluble sugar, soluble protein and proline in *P. vulgaris*, which helped to maintain the osmotic balance in cells, and reduced the damage to the plasma membrane and the content of ROS in coordination with antioxidant enzymes. Similar results were also found in maize (*Zea mays*) [15] and sugar beet (*Beta vulgaris*) [48].

Compared with the control treatment, MT treatments elevated the chlorophyll content in *P. vulgaris* leaves. In *B. juncea* [44], *Camellia sinensis* [49], and *Triticum aestivum*, the application of MT enhanced the chlorophyll content [50], and these results supported our findings. The increase in chlorophyll by MT may be achieved by up-regulating chlorophyll biosynthesis genes to promote the accumulation of chlorophyll content, and by inhibiting the expression of chlorophyll degradation enzymes to delay the degradation of chlorophyll [49,51]. Moreover, carotenoids can not only absorb and transform light energy, but also quench ROS to reduce the intensity of oxidative stress and protect chlorophyll from photooxidation damage [52,53]. So, the increase in the Car content in *P. vulgaris* might be one of the reasons for the higher chlorophyll content after MT treatment.

MT application (100 μM) greatly promoted the increase in the stomatal opening alone with G_s_, resulting in more CO_2_ production in intercellular spaces, and thus improving CO_2_ assimilation efficiency [44]. In this study, P_n_, G_s_, C_i_, and T_r_ all increased after MT treatments, indicating that MT could improve stomatal function and increase the accumulation of intracellular CO_2_, thus increasing the photosynthetic capacity of *P. vulgaris* leaves [45]. The application of MT in *B. juncea* [44] and *C. sinensis* [49] also produced similar results. Water use efficiency is closely related to the short-term regulation of stomatal aperture [54]. In transgenic apples, the overexpression of *MdASMT9* increased MT biosynthesis, and improved water use efficiency by increasing the photosynthetic rate and stomatal opening [55], which was also observed in our study (data not shown).

The characteristics of root architecture and physiology are closely related to the absorption of fertilizer and water by plants. The root length reflects the extension range of the root system, the vertical extension of the root system is conducive to the absorption of water and nutrients in the deep soil by plants, and the total root volume reflects the robustness of the plant anchoring [56]. The root surface area reflects the contact area between plants and soil, which is closely related to the absorption of water and nutrients by roots, and the number of root tips and branches reflects the absorption capacity of roots [57]. In this study, exogenous applications of MT obviously improved the root architecture of *P. vulgaris*, which would help to promote the absorption of water and nutrients by roots, and promoted the synthesis of carbohydrates and the accumulation of dry matter [58]. Studies showed that genes and networks related to the IAA pathway were regulated by MT at the transcription and post-transcription level [19,20], and a MT receptor (CAND2/PMTR1) was proved to be involved in the regulation of root growth by bacterial N-acyl-homoserine lactones [21,22,23,24]. These studies suggested that MT could regulate multiple signal pathways to regulate the root growth of *P. vulgaris*. In this study, the low concentrations of 50–200 μM MT promoted root growth, while the high concentration of 400 μm MT inhibited the root growth of *P. vulgaris*. Similar results were also found in *B. juncea* [59] and *Cyamopsis tetragonoloba* [60], indicating that MT affects root growth and development in a dose-dependent manner.

The relative variable fluorescence (W_K_) at the K point reflects the inhibition of the oxygen-evolving complex (OEC) on the donor side of the PSII reaction center. The increase in variable fluorescence at the J point reflects the electron blockage between the quinone acceptor Q_A_ and Q_B_. M_0_ refers to the rate at which the primary quinone acceptor (Q_A_) is reduced and reflects the maximum rate of closure of the PSII reaction center, and φE_0_ reflects the quantum of light energy absorbed by the reaction center for electron transfer production [61]. The study showed that MT application (100 μM) decreased the variable fluorescence at the K point (W_K_) and J point (V_J_) in the OJIP curve, and M_0_ decreased while φE_0_ augmented. These indicated that the application of MT improved the activity of OEC, reduced the maximum closing rate of the PSII reaction center, and elevated the electron transfer capacity of the PSII donor side and receptor side in *P. vulgaris* [14]. The blockage of electron transport resulted in electron leakage during electron transfer, which would attack O_2_ to generate ROS and cause membrane lipid peroxidation [62]. Conversely, the improvement of the electron transport capacity of PSII after 100 μM MT treatment inevitably reduced the levels of ROS and membrane lipid peroxidation in *P. vulgaris* leaves and protected the stability of the cell membrane.

In this study, the energy absorption (ABS/CS_m_), capture (TR_0_/CS_m_), electron transfer (ET_0_/CS_m_), and heat dissipation (DI_0_/CS_m_) per cross section in the 100 μM MT treatment were all greatly higher than those in the control treatment. Except for DI_0_/CS_m_, which was slightly higher in the 100 μM MT treatment than in the control, the other three parameters in the treated leaves were much higher than those of the control. However, the absorption (ABS/RC), capture (TR_0_/RC), electron transfer (ET_0_/RC), and heat dissipation (DI_0_/RC) per reaction center in the 100 μM MT treatment were all decreased compared to those in the control treatment. Furthermore, the 100 μM MT treatment significantly elevated the number of active reaction centers per cross section (RC/CS_m_) of PSII. These showed that the 100 μM MT treatment could greatly decrease the energy charge pressure of PSII reaction to reduce the occurrence of photoinhibition, and improve the energy conversion and utilization efficiency by increasing the RC/CS_m_ of PSII [63,64].

F_v_/F_m_ refers to the maximum photochemical efficiency of PSII; PI_abs_ represents the performance index on absorption basis, and includes three aspects of light energy absorption, capture, and electron transfer, which can comprehensively reflect the performance of PSII [65]. In this study, the parameters of RC/CS_m_, F_v_/F_m_ and PI_abs_ by 100 μM MT were enhanced in *P. vulgaris*. Similar results were also found in *Vigna angularis* [66] and *Chara australis* [64] under the appropriate MT treatments. The increase in F_v_/F_m_ and PI_abs_ indicated that 100 μM MT could provide maximum light energy for the photosynthetic electron transport chain, and increased the maximum photochemical efficiency of PSII and the photosynthetic activity of PSII in *P. vulgaris*, which was also confirmed by the increase in the ABS/CS_m_, Tr_0_/CS_m_, ET_0_/CS_m_, and P_n_ of leaves after the 100 μM MT treatment. *P. vulgaris* had higher root absorption and photosynthetic capacities under the suitable MT treatments, which explained the higher dry mass of the whole plant and spica under these treatments.

The 820-nm modulated reflectance curve represents the redox activity of PSI; the lower the minimum MR/MR_0_, the stronger the ability of the PSI reaction center to reduce the terminal electron acceptor [63]. The variable ΔI/I_0_ reflects the maximum redox capacity of the PSI reaction center P700 and is used to comprehensively evaluate the performance of PSI; Φ_PSI/PSII_ (ΔI/I_0_/ψ_0_) is used to characterize the coordination between PSII and PSI [67]. The minimum value of MR/MR_0_ greatly decreased, and the values of ΔI/I_0_ and Φ_PSI/PSII_ markedly enhanced after 100 μM MT treatment. These indicated that 100 μM MT greatly increased the activity of the PSI reaction center, elevated the mobility of electrons from PSII to PSI, and improved the coordination between both the photosystems.

Many studies have shown that flavonoids and phenolic components are secondary metabolites with antioxidant capabilities in plants and are also important components of non-enzymatic antioxidants in plants, which are another line of defense in the scavenging process of ROS [4,12,68]. In the present study, 100 μM MT significantly enhanced the antioxidant capacity via promoting the accumulation of total flavonoids and phenolic components in *P. vulgaris*. Caffeic acid is a naturally occurring hydroxycinnamic acid phenol in many plants, such as coffee and tea, and has been proven to have antioxidant, anti-inflammatory, cardiovascular protective, and hypoglycemic activities [69]. Ferulic acid is a phenolic acid with antithrombotic, anti-inflammatory, anticancer, and antioxidant activities [70]. As an important phenolic component, rosmarinic acid is used as a standard to measure the quality of *P. vulgaris* in Chinese Pharmacopoeia; it also has good scavenging ability against ROS [4]. Hyperoside is also a ROS scavenger; it has been shown to increase the activity of glutathione peroxidase and inhibit the H_2_O_2_-induced apoptosis of Chinese hamster lung fibroblasts [4]. Therefore, the increase in the above four compounds might contribute to improving the antioxidant capacity of *P. vulgaris*. In this study, the increase in the contents of total flavonoids, total phenolics, caffeic acid, ferulic acid, and rosmarinic acid might be due to the signal function of MT, which stimulates the biosynthesis of secondary metabolites by inducing various physiological and metabolic pathways of plant hormones [18]. Previous studies have also shown that MT can induce the expression of flavonoid biosynthesis genes and related transcription factors [68], promote the upregulation of genes related to phenolic acid synthesis such as *PAL*, *STS*, *C4H*, and *CHS* [12], and enhance the expression of genes related to rosmarinic acid synthesis such as *PAL* and *RAS* genes [71]. In line with these results obtained in the present investigation, the application of MT has also been shown to increase the contents of phenolic compounds, caffeic acid, and rosmarinic acid in the callus of *R. officinalis* [8], and the contents of phenolics and rosmarinic acid in the callus of *O. basilicum* [72].

## 4. Materials and Methods

### 4.1. Plant Materials

The seeds of *P. vulgaris* were disinfected with 2% sodium hypochlorite, washed with clean water three times, and then planted in the experimental field in October 2021. In April 2022, seedlings of the same growth were transplanted into pots (26 cm × 22 cm, height × diameter) filled with soil and peat soil (*v*/*v* = 3:1), and four seedlings were planted in each pot. The seedlings were cultured in a growth room with a light intensity of 900 μmol·m^−2^·s^−1^, photoperiod of 12 h, temperature of 26 /20 °C (day/night), relative humidity of 70% ± 5%, under normal water management.

### 4.2. Experimental Design

Pot culture was carried out to see the effect of exogenous MT on *P. vulgaris*. MT was first dissolved in ethanol and then diluted to five concentrations such as 0 (CK), 50, 100, 200, and 400 μM, respectively, where CK was the control containing no MT but deionized water. For each treatment level of MT concentration, 10 replicate pots were used. So, a total of 50 pots were prepared with *P. vulgaris* for the experiment. Besides MT, a 0.2% KH_2_PO_4_ solution was also sprayed. In May 2022, the treatment was started at the flowering stage of *P. vulgaris* and continued for six consecutive days. Starting with the first day of the experiment, the respective concentration of MT was sprayed to each of the four groups of treatment plants and continued for each one-day interval. The plants of the control group were always sprayed with the same amount of deionized water. While starting from the second day, the experimental plants from all experimental groups received spraying with the 0.2% KH_2_PO_4_ solution and continued at each one-day interval for foliar fertilization of all treatment groups. So, the experimental plants had received an alternating MT or 0.2% KH_2_PO_4_ solution for a total of three days, respectively, out of a six-day treatment duration. Foliar spraying with MT and 0.2% KH_2_PO_4_ solution was carried out around 6:00 P.M. on each occasion, subject to the leaves being covered with droplets. After the treatment was complete, the experimental plants were allowed to grow for 2 weeks. Thereafter, photosynthetic and fluorescence indexes were determined, and the biochemical indexes of *P. vulgaris* were measured. *P. vulgaris* seedlings were harvested in late June, and dried to a constant weight in an oven at 50 °C to determine the dry mass. The dried spicas were powdered and passed through a 60-mesh sieve. Then, the bioactive components were determined using high-performance liquid chromatography (HPLC).

### 4.3. Determination of Antioxidant Enzyme Activity

Fresh leaves (0.20 g) of *P. vulgaris* were ground into powder with liquid nitrogen, and then 0.05 mM phosphate-buffered saline (PBS, pH 7.8) was added to the ice bath to prepare the homogenate. The homogenate was centrifuged at 3000 rpm for 10 min at 4 °C; the supernatant was used for determining superoxide dismutase (SOD) and peroxidase (POD) enzyme activities, and the results were expressed as U g^−1^ min^−1^ FW. The SOD enzyme activity was determined as described by Li et al. [73]. The 200 μL extract was mixed with 0.05 mM PBS, ethylenediaminetetraacetic acid disodium salt (100 mM), L-methionine (130 mM), nitroblue tetrazolium (NBT) (750 μM), and riboflavin (20 μM) to form a 3 mL reaction solution. The absorbance of the mixture at 560 nm was measured using a spectrophotometer (752N, INESA, Shanghai, China) after irradiation under 50 μmol m^−2^ s^−1^ for 20 min, and the inhibition of NBT photoreduction by 50% was taken as the unit of SOD enzyme activity. The POD activity was measured as described by Li et al. [74]. The reaction solution mixture comprised 50 mM PBS (pH 6.0), 30% H_2_O_2_ (*v*/*v*), 100% guaiacol solution, and 1 mL enzyme extract. The POD activity was calculated by measuring the changes in absorbance at 470 nm.

### 4.4. Determination of Soluble Sugar Content

The fresh leaves from the different treatments were immersed in a test tube containing 8 mL of distilled water. The samples were extracted twice in boiling water for 30 min and then filtered into a volumetric flask. Then, 0.5 mL of the extract was mixed with 1.5 mL of deionized water, 0.5 mL of ethyl anthracene acetate, and 5 mL of concentrated sulfuric acid. The mixture was boiled for 1 min. According to the method of Dong et al. [75], the absorbance of the extract at 630 nm (X) was measured using a spectrophotometer. The soluble sugar content (Y) was calculated according to the standard curve of glucose (Y = (X − 0.0057)/0.0047).

### 4.5. Determination of Photosynthetic Pigment

The contents of chlorophyll a (Chl a, C_a_), Chl b (C_b_) and carotenoid (Car, C_car_) were measured by the method proposed by Lichtenthaler [76]. The leaf samples (0.20 g) were suspended in 10 mL of 80% acetone in the dark for 48 h, and the absorbance of the extract at 470, 646, and 663 nm was determined using a spectrophotometer, the photosynthetic pigments were calculated according to the following formulae:

C_a_ (mg/g FW) = (12.21 × A_663_ − 2.81 × A_646_) × V/W,

C_b_ (mg/g FW) = (20.13 × A_646_ − 5.03 × A_663_) × V/W,

C_car_ (mg/g FW) = ((1000 × A_470_ − 3.27 × C_a_ − 104 × C_b_)/229) × V/W.

### 4.6. Determination of Proline Content

The proline content was determined as proposed by Bates et al. [77]. At first, 0.25 g leaves were homogenized in 5 mL of 3% aqueous sulfosalicylic acid and boiled for 10 min. The supernatant (2 mL) was reacted with 2 mL of acetic acid and 2 mL of acid ninhydrin in a test tube for 30 min at 100 °C. Afterwards, the reaction mixture was cooled, and 4 mL of toluene was added to complete extraction. The absorbance of the toluene layer was measured at 520 nm (X) using a spectrophotometer. The content of proline (Y) in the sample was calculated according to the standard curve (Y = (X − 0.0011)/0.0525), and the results were expressed as µg g^−1^ FW.

### 4.7. Measurement of Malondialdehyde Content

The malondialdehyde (MDA) content was measured as described by Cai et al. [78]. Fresh leaves (0.25 g) were ground to make powder with liquid nitrogen, and 5 mL of trichloroacetic acid (5%, *w*/*v*) was added to prepare a homogenate, which was centrifuged at 3000 rpm for 10 min to collect the supernatant. Then, 2 mL of the supernatant was mixed with 2-thiobarbituric acid (0.67%, *w*/*v*) and incubated at 100 °C for 30 min. The absorbance of the supernatant at 450, 532, and 600 nm was recorded using a spectrophotometer. The content of MDA was calculated according to the formula (C (µmol g^−1^) = 6.45 × (A_532_ − A_600_) − 0.56 × A_450_).

### 4.8. Determination of Relative Electrical Conductivity

The relative electrical conductivity (REC) of the leaves was determined as described by Zhang et al. [67]. Fresh leaves (0.10 g) were cut into strips, rinsed with deionized water, and placed in test tubes with 5 mL of deionized water. The samples were placed at room temperature in the dark for 24 h, and their initial electrical conductivity (C1) was measured. Then, the test tubes were placed in boiling water for 30 min to obtain the maximum electrical conductivity (C2) after cooling. The REC was calculated based on the percentage of C1/C2 × 100%.

### 4.9. Determination of Hydrogen Peroxide Content

The hydrogen peroxide (H_2_O_2_) content was measured according to the method described by Alexieva et al. [79]. Fresh leaf samples (0.5 g) were quickly ground into a powder with liquid nitrogen. The homogenate was obtained by grinding in an ice bath after adding 5 mL of trichloroacetic acid (1% *w*/*v*), and then centrifuged at 12,000 rpm at 4 °C for 20 min. The supernatant (0.7 mL) was mixed with 0.7 mL of 10 mM phosphate buffer (pH 7.0) and 1.4 mL of 1 M potassium iodide. After 1 h of reaction in the dark, the absorbance of the supernatant at 390 nm (X) was measured using a spectrophotometer. The H_2_O_2_ content (Y) was calculated according to the standard curve (Y = (X + 0.0187)/0.0136), and the results were represented as µmol g^−1^ FW.

### 4.10. Root Morphology

The roots were cut from the seedlings and washed with deionized water, and then the root morphology after different treatments was examined using a scanner (Epson Expression 12000XL, Seiko Epson Co., Ltd. Tokyo, Japan). The total root length, -surface area, -volume, -tip number, and -branch number were measured using the WinRHIZO Root Analysis System (Regent Instruments, Sainte Foy, QC, Canada). A total of 10 seedlings were measured for each treatment.

### 4.11. Determination of Dry Mass

Ten plants per biological replicate were randomly selected for each assay. The plants were dried at 50 °C for 7 days for the biomass measurements, including the dry mass of the whole plant and spica.

### 4.12. Determination of Gas Exchange Parameters

Gas exchange parameters including net photosynthetic rate (P_n_), intercellular CO_2_ concentration (C_i_), stomatal conductance (G_s_), and transpiration rate (T_r_) were measured using an LI-6400 portable photosynthetic system (LI-COR, Lincoln, NE, USA) from 9:00–10:00 A.M. on sunny days. An internal light-emitting diode light source was used to adjust the saturated photosynthetic photon flux density to 1000 μmol m^−2^ s^−1^. The leaf chamber temperature was set to 25 °C. An air flow rate of 500 μmol s^−1^ was used to obtain CO_2_ from the environmental source, and the CO_2_ concentration was maintained at 380 μmol mol^−1^ using soda lime. All treatments were conducted for five biological repeats, and three leaves were selected for each biological repeat.

### 4.13. Fast Chlorophyll Fluorescence and 820 nm Modulated Reflection

M-PEA (Hansatech, Norfolk, UK), a multifunctional plant efficiency analyzer, was used simultaneously to measure the fast fluorescence OJIP curve and 820 nm modulated reflection curve of *P. vulgaris* leaves [65]. Before the measurement, the leaves were dark-adapted for at least 30 min and then exposed to saturated red light (3000 μmol m^−2^ s^−1^) for 1 s. The chlorophyll fluorescence signal was continuously recorded to obtain the fast fluorescence OJIP curve. The modulated reflection curve at 820 nm was measured by simultaneous exposure to 250 μmol m^−2^ s^−1^ far-red light as described by Strasser et al. [65]. The following parameters were calculated according to the analysis of the JIP test (Table 4). The relative variable fluorescence (V_t_) at any time was calculated as: V_t_ = (F_t_ − F_0_)/(F_m_ − F_0_), based on the method proposed by Li et al. [29]. The 820-nm modulated reflection curve was plotted according to its relative value (MR/MR_0_), where MR is the modulated reflection at different time points, and MR_0_ is the MR value of far-red light irradiated at 0.7 ms [65]. The maximum redox activity of PSI and the coordination between PSII and PSI were calculated according to the 820 nm modulated reflection curve: ΔI/I_0_ = (I_0_ − I_m_)/I_m_, Φ_(PSII/PSII)_ = (ΔI/I_0_)/ψ_0_ [67,80]. Ten replicates were measured for each treatment.

### 4.14. Determination of Total Phenolic Content

The total phenolic content of *P. vulgaris* spicas was determined according to the method of Zhu et al. [81]. Dried spica powder (0.2 g) was added to 10 mL of 30% ethanol and extracted by ultrasound at 50 °C for 30 min. Then the extract (0.5 mL) was mixed with 2.5 mL of 10% Folin Ciocalteu’s reagent and 2 mL of 7.5% (*w/v*) Na_2_CO_3_. After the mixture was shaken well, the absorption value was determined at 765 nm (X) using a spectrophotometer after 60 min in the dark at room temperature. The content of phenolic compounds (Y) was calculated by the standard correction curve (Y = (X − 0.214)/0.0798) drawn by gallic acid.

### 4.15. Determination of Total Flavonoids Content

The content of total flavonoids was determined using the method proposed by Chen et al. [4]. The dried spica powder (0.2 g) was mixed with 10 mL of a 35% ethanol solution and extracted three times in a water bath at 86 °C for 3.5 h. Then, 1 mL of the extract was mixed with 0.3 mL of 5% NaNO_2_ and reacted for 6 min. Next, 0.3 mL of 10% Al(NO_3_)_3_ solution was mixed with the reaction solution for 6 min. Finally, 4.0 mL of 4% NaOH was added and mixed, and the absorbance was measured at 510 nm (X) after 15 min using a spectrophotometer. The content of total flavonoids (Y) was calculated by the standard correction curve (Y = (X + 0.0301)/11.688) generated by rutin.

### 4.16. Caffeic Acid, Ferulic Acid, Rosmarinic Acid, and Hyperoside Contents

The contents of caffeic acid, ferulic acid, rosmarinic acid, and hyperoside were determined using a previously described method with minor modifications [4]. Dried spica powder (0.2 g) was extracted ultrasonically by adding 20 mL of 80% methanol (containing 1% formic acid) for 30 min at 50 °C, and the extract was centrifuged at 10,000 rpm for 10 min. The supernatant was filtered with a 0.22 μm organic membrane filter for HPLC analysis. The extracts were quantified using a HPLC instrument (Agilent 1260, Agilent Technology Co., Ltd., Santa Clara, CA, USA) equipped with a diode array detector and Waters C18 column (250 mm × 4.6 mm).

Methanol (A) and sodium dihydrogen phosphate solution (0.2%, B) were used as the mobile phase for gradient elution. The elution program was as follows: 0–20 min, 20–40% A; 20–35 min, 40–70% A; 35–45 min, 70–90% A; 45–60 min, 90–20% A; injection volume 20 μL; flow rate 0.8 mL min^−1^; column temperature 30 °C. Caffeic acid, ferulic acid, rosmarinic acid, and hyperoside were determined at 325 nm. Each peak was identified by comparing the relative retention time according to the signal spectrum of the standard. The contents of caffeic acid (Y = (X − 72.87)/4458.00), ferulic acid (Y = X + 67.67)/4704.1), rosmarinic acid (Y = (X − 242.04)/3398.70) and hyperoside (Y = (X − 23.24)/1944.90) were calculated according to the standard curve of the reference substances; Y was the concentration of each substance, and X was the peak area of the sample. The results were represented as mg g^−1^.

### 4.17. Statistical Analysis

A one-way analysis of variance and Duncan test (*p* < 0.05) were performed using SPSS 13.0 software (SPSS, New York, IL, USA). The data were expressed as mean ± standard deviation. At least three biological replicates were used to determine the biochemical parameters.

## 5. Conclusions

In this study, the 100 μM treatment of MT greatly improved the antioxidant capacity by increasing the activities of antioxidant enzymes and the contents of osmoregulation substances, carotenoids, flavonoids and polyphenols, markedly reduced the content of ROS, and enhanced the stability of cell membrane in *P. vulgaris*. MT significantly stimulated the development of the root system and improved its absorption capacity. The photosynthetic capacity of *P. vulgaris* leaves considerably improved through the increase in the activities and coordination of PSII and PSI, as well as the photosynthetic pigment contents. Furthermore, the dry mass and secondary metabolite contents of *P. vulgaris* were drastically improved. Our results demonstrated that MT increased the yield and medicinal quality, through regulating antioxidant metabolism, root growth and photosynthetic capacity. The concentration of 100 μM proved to be the best among the various concentrations tested, which can be considered for the production of *P. vulgaris*.

## Figures and Tables

**Table 1:** Changes in root architecture of *P. vulgaris* under different MT treatments.

MT (μM)	Root Length (cm)	Root Surface Area (cm^2^)	Root Volume (cm^3^)	The Number of Root Tip	Branch Number
0	628.52 ± 6.28 d	71.08 ± 11.93 d	0.54 ± 0.03 c	1185.67 ± 101.16 d	1703.00 ± 64.09 c
50	791.76 ± 21.14 c	89.79 ± 0.94 bc	0.81 ± 0.02 bc	1594.67 ± 41.48 b	2533.67 ± 63.00 a
100	982.88 ± 43.78 a	119.08 ± 4.83 a	1.15 ± 0.04 a	1989.00 ± 86.85 a	2662.00 ± 127.47 a
200	927.60 ± 2.77 b	100.60 ± 0.61 b	0.87 ± 0.02 b	1344.00 ± 32.60 c	2331.67 ± 20.58 b
400	603.97 ± 19.48 d	79.19 ± 3.77 cd	0.83 ± 0.05 bc	978.00 ± 13.08 e	1396.67 ± 100.78 d

MT: melatonin. Data are means ± standard error; different letters indicate significant differences as determined by the Duncan test (*p* ≤ 0.05).

**Table 2:** The changes in energy fluxes in *P. vulgaris* leaves under different MT treatments (μM).

Parameter	0	50	100	200	400
ABS/RC	1.88 ± 0.09 a	1.69 ± 0.07 ab	1.49 ± 0.12 c	1.55 ± 0.08 c	1.78 ± 0.20 a
DI_0_/RC	0.47 ± 0.04 a	0.40 ± 0.04 ab	0.33 ± 0.06 c	0.34 ± 0.03 c	0.45 ± 0.06 a
Tr_0_/RC	1.41 ± 0.05 a	1.29 ± 0.03 abc	1.15 ± 0.06 c	1.20 ± 0.06 bc	1.33 ± 0.15 ab
ET_0_/RC	0.66 ± 0.03 a	0.60 ± 0.05 a	0.62 ± 0.06 a	0.63 ± 0.07 a	0.57 ± 0.10 a
RC/CS_m_	10875.49 ± 613.14 c	14115.10 ± 479.54 b	16826.63 ± 2153.49 a	15545.80 ± 525.87 ab	11392.45 ± 1171.21 c
ABS/CS_m_	24516.67 ± 284.63 b	28553.33 ± 1521.33 a	29790.67 ± 1597.43 a	28850.67 ± 2260.85 a	24189.00 ± 502.50 b
DI_0_/CS_m_	6091.00 ± 171.47 a	6755.67 ± 644.11 a	6572.67 ± 408.29 a	6417.00 ± 628.87 a	6085.33 ± 178.07 a
Tr_0_/CS_m_	18425.67 ± 456.11 b	21797.67 ± 1035.44 a	23218.00 ± 1910.84 a	22433.67 ± 1685.98 a	18103.67 ± 556.01 b
ET_0_/CS_m_	8614.67 ± 132.21 cd	10234.33 ± 1200.04 bc	12352.67 ± 826.32 a	11677.67 ± 1578.10 ab	7743.67 ± 661.03 d

MT: melatonin. Data are means ± standard error; different letters indicate significant differences as determined by the Duncan test (*p* ≤ 0.05).

**Table 3:** Effects of different concentrations of MT on the contents of total flavonoids, total phenolics, caffeic acid, ferulic acid, rosmarinic acid, and hyperoside in *P. vulgaris* (mg/g).

MT (μM)	Total Flavonoids	Total Phenols	Caffeic Acid	Ferulic Acid	Rosmarinic Acid	Hyperoside
0	80.53 ± 1.02 d	18.54 ± 0.73 e	0.17 ± 0.00 c	0.31 ± 0.00 b	5.65 ± 0.07 b	0.45 ± 0.02 c
50	85.92 ± 0.59 c	19.96 ± 0.74 d	0.18 ± 0.01 b	0.32 ± 0.01 b	6.11 ± 0.09 a	0.52 ± 0.02 b
100	100.56 ± 1.33 a	32.02 ± 0.56 a	0.21 ± 0.00 a	0.35 ± 0.00 a	6.51 ± 0.36 a	0.64 ± 0.03 a
200	91.74 ± 1.41 b	25.83 ± 0.41 b	0.22 ± 0.00 a	0.35 ± 0.01 a	6.21 ± 0.10 a	0.54 ± 0.03 b
400	86.80 ± 0.89 c	22.07 ± 0.81 c	0.19 ± 0.01 b	0.32 ± 0.01 b	5.42 ± 0.30 b	0.53 ± 0.02 b

MT: melatonin. Data are means ± standard error; different letters indicate significant differences as determined by the Duncan test (*p* ≤ 0.05).

**Table 4:** Parameters in JIP test analysis.

Fluorescence Parameters	Description
W_K_ = (F_K_ − F_0_)/(F_J_ − F_0_)	Normalized relative variable fluorescence
V_J_ = (F_J_ − F_0_)/(F_m_ − F_0_)	Relative variable fluorescence intensity at the J step
M_0_ = 4 (F_300μs_ − F_0_)/(F_m_ − F_0_)	Initial slope of the relative variable fluorescence of the relative rate at which *Q*_A_ is reduced
φE_0_ = ET_0_/ABS = [1− (F_0_/F_m_)]ψ_0_	Quantum yield for electron transport
ABS/RC = M_0_ (1/V_J_) (1/φP_0_)	Absorption flux per reaction center
TR_0_/RC = M_0_(1/V_J_)	Trapped energy flux per RC
ET_0_/RC = M_0_ (1/V_J_) ψE_0_	Electron transport flux per RC
DI_0_/RC = (ABS/RC) − (TR_0_/RC)	Dissipated energy flux per RC
RC/CS_m_ = φP_0_ (V_J_/M_0_) (ABS/CS_m_)	Density of RCs per excited cross section (CS)
ABS/CS_m_ ≈ F_m_	Absorbed energy flux per CS
TR_0_/CS_m_ = φP_0_(ABS/CS_m_)	Trapped energy flux per CS
ET_0_/CS_m_ = φE_0_(ABS/CS_m_)	Electron transport flux per CS
DI_0_/CS_m_ = ABS/CS_m_-TR_0_/CS_m_	Dissipated energy flux per CS
F_v_/F_m_	Maximal quantum yield of PSII photochemistry
PI_ABS_ = (RC/ABS) [φP_0_/(1 − φP_0_)][ψ_0_/(1 − ψ_0_)]	Performance index on absorption basis

## Data Availability

Not applicable.
